# Supplementary material for: Stacks off tracks: a role for the golgin AtCASP in plant endoplasmic reticulum-Golgi apparatus tethering
Source: J Exp Bot. 2017 Jun 9;68(13):3339–50. doi: 10.1093/jxb/erx167 (PMC5853478; doi:10.1093/jxb/erx167)
Supplement: Supplementary Data Video Legends [file erx167_suppl_supplementary_data_video_legends.pdf]

Supplementary Data: video legends

Stacks off tracks: A role for the golgin AtCASP in plant endoplasmic reticulum-Golgi apparatus tethering

Anne Osterrieder[1]*, Imogen A Sparkes[1,2], Stan W Botchway[3], Andy Ward[3], Tijs Ketelaar[4], Norbert de Ruijter[4], and Chris Hawes[1]*

[1] Department of Biological and Medical Sciences, Faculty of Health and Life Sciences, Oxford Brookes University, Gipsy Lane, Headington, Oxford, OX3 0AZ, UK.

[2] Present address: Biosciences, College of Life and Environmental Sciences, Geoffrey Pope, University of Exeter, Exeter, EX4 4QD, UK

[3] Central Laser Facility, Science and Technology Facilities Council, Research Complex at Harwell, Didcot, Oxon OX11 0FA, UK

[4] Laboratory of Cell Biology, Wageningen University, Droevendaalsesteeg 1, 6708PB Wageningen, The Netherlands.

*Correspondence: a.osterrieder@brookes.ac.uk and Chawes@brookes.ac.uk

Received 14 Mar 2017; Accepted 25 April 2017

Supplementary Video S1: confocal images of a 34.4 s time series showing Arabidopsis leaf epidermal cells with ER labelled with GFP-HDEL and Golgi bodies labelled with mRFP- AtCASP-ΔCC.

Initially, a whole group of Golgi bodies moved with the trap. A single Golgi body remained in the trap, lost connection to the ER tubule, and then moved freely through the cell until connection was re-established near an ER tubule. Scale bar=2 μm.

Supplementary Video S2: confocal images of a 70.4 s time series showing Arabidopsis leaf epidermal cells with ER labelled with GFP-HDEL and Golgi bodies labelled with mRFP-AtCASP-ΔCC.

The optically trapped Golgi body lost its connection to the ER, its movement being mirrored by the ER with a gap between the both. Scale bar=2 μm.

Supplementary Video S3: confocal images of a 15.24 s time series showing Arabidopsis leaf epidermal cells with ER labelled with GFP-HDEL and Golgi bodies labelled with STtmd-mRFP.

The movement of the trapped Golgi body and the tip of the ER tubule overlaid almost perfectly with each other during micromanipulation. Scale bar=2 μm.

Supplementary Video S4: confocal images of a 10.40 s time series showing Arabidopsis leaf epidermal cells with ER labelled with GFP-HDEL and Golgi bodies labelled with mRFP-AtCASP.

Golgi and ER tubule tracks mirrored each other as in the control, but the ER-Golgi connection was more easily disrupted. Scale bar=2 μm.

Supplementary Video S5: confocal images of a 10.27 s time series showing Arabidopsis leaf epidermal cells with ER labelled with GFP-HDEL and Golgi bodies labelled with mRFP-AtCASP-ΔCC.

The ER tubule initially followed the trapped Golgi body. The connection became disrupted, but the ER continued to mirror the Golgi body movement. Scale bar=2 μm.
